# Left pulmonary artery pseudoaneurysm secondary to post‐operative lung abscess

**DOI:** 10.1002/rcr2.598

**Published:** 2020-06-16

**Authors:** Dario Amore, Giorgio Bocchini, Dino Casazza, Umberto Caterino, Albina Palma, Carlo Curcio

**Affiliations:** ^1^ Department of Thoracic Surgery Monaldi Hospital Naples Italy; ^2^ Department of Diagnostic Imaging, General Radiology Monaldi Hospital Naples Italy; ^3^ Thoracic Endoscopic Unit Monaldi Hospital Naples Italy; ^4^ Emergency Department C.T.O. Hospital Naples Italy

**Keywords:** Critical care medicine, infection and inflammation, thoracic surgery

## Abstract

A prompt diagnosis is mandatory to avoid fatal complications in case of pulmonary artery pseudoaneurysm.

## Graphical abstract

We report a rare case of post‐operative pulmonary artery pseudoaneurysm. In such instance a prompt diagnosis and adequate treatment are essential to avoid catastrophic complications.

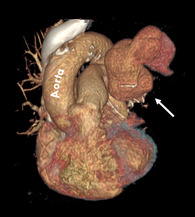

## Clinical Image

**Figure 1:**
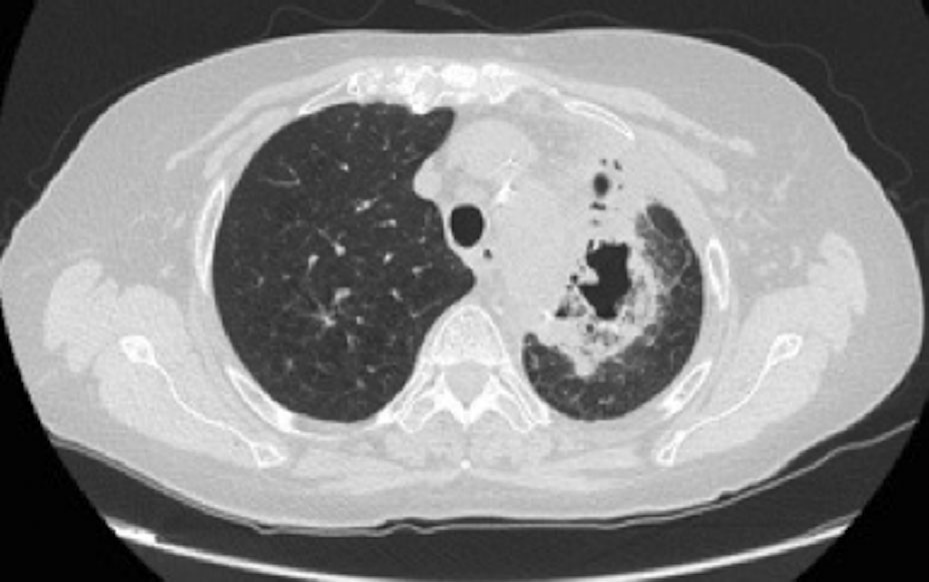
Cavitary left lung lesion detected on post‐operative chest computed tomography.

**Figure 2:**
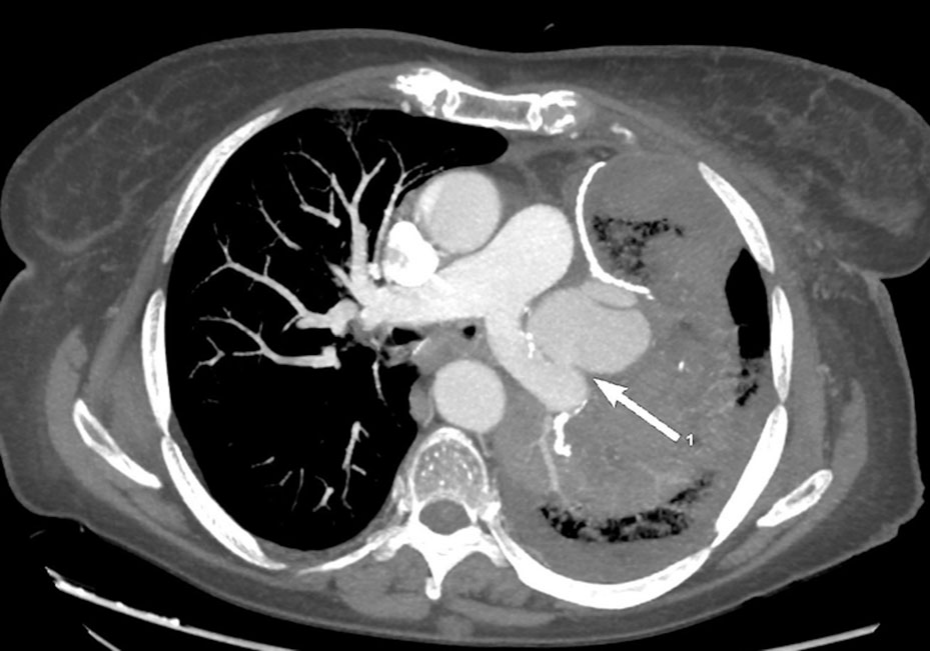
Axial chest computed tomography angiography shows pulmonary artery pseudoaneurysm (white arrow) surrounded by lung parenchymal consolidation.

**Figure 3:**
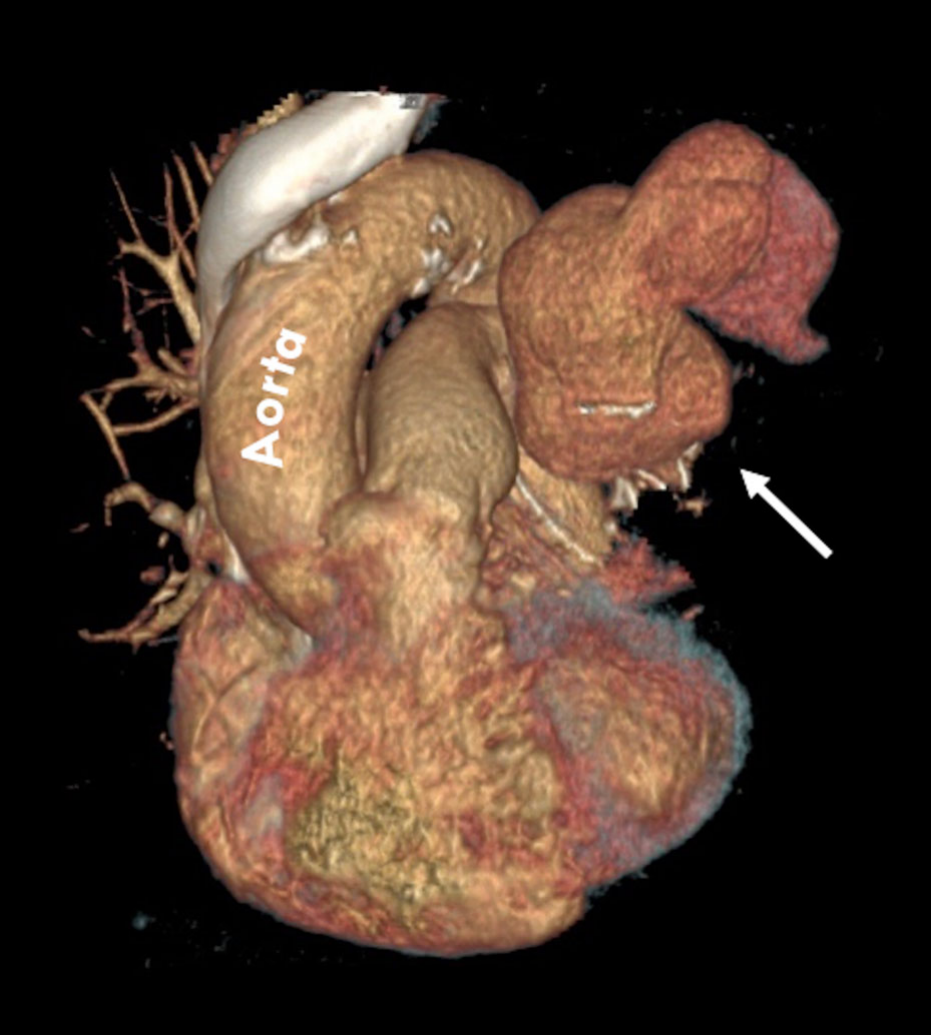
Computed tomography angiography with three‐dimensional reconstruction shows a large contrast‐filled outpouching adjacent to the left pulmonary artery (white arrow).

**Figure 4:**
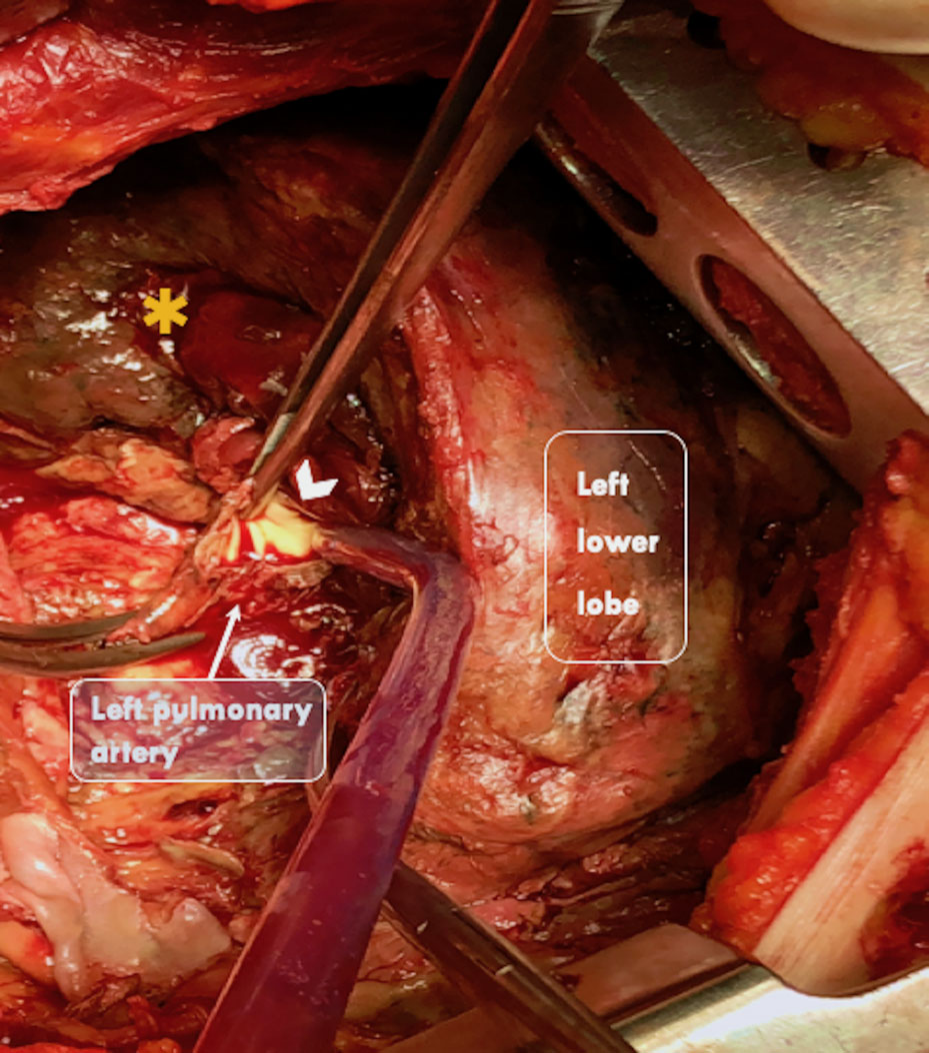
Intra‐operative view: necrotic lung tissue (orange asterisk) of the remnant left lower lobe and rupture involving the distal left pulmonary artery (white arrow head).

A 68‐year‐old woman was admitted to our unit for treatment of lung adenocarcinoma and scheduled for thoracoscopic left upper lobectomy. She had an uneventful hospital stay and was discharged on the fifth post‐operative day. After 24 h, the patient experienced a high fever and she was admitted to the hospital. Post‐operative chest computed tomography (CT) revealed a lung abscess in the left lower lobe (Fig. 1). Since the patient, in the following hours, began experiencing haemoptysis, a bronchoscopy was carried out but no active source of bleeding or bronchopleural fistula was identified. However, a chest CT angiography showed extravasation of contrast medium from the left pulmonary artery (Figs. 2, 3). Due to uncontrolled haemoptysis and extensive left lung consolidation, an urgent left lateral thoracotomy was performed and a pulmonary artery rupture was identified (Fig. 4). Since the interlobar artery was friable and the mobilization of residual lower lobe caused an extension of the vascular rupture, a completion pneumonectomy was performed. *Moraxella catarrhalis* was isolated from sputum and tissue cultures. Post‐operative pulmonary artery pseudoaneurysm is a rare event described in only few case reports [1, 2]. In such instance a prompt diagnosis and adequate treatment are essential to avoid catastrophic complications.

### Disclosure Statement

Appropriate written informed consent was obtained for publication of this case report and accompanying images.
